# Genome-Wide Association Analysis of Sweet Pepper (*Capsicum annuum*) Based on Agronomic Traits Using PepperSNP50K

**DOI:** 10.3390/plants14101506

**Published:** 2025-05-17

**Authors:** Yaolong Wang, Entong Li, Jiawei Lu, Jing Wang, Qiaolu Zang, Yanping Liang, Ruxia Tian, Changwei Zhang, Fangling Jiang, Yan Cheng

**Affiliations:** 1 Horticulture College, Shanxi Agricultural University, Taiyuan 030032, China; 2022104074@stu.njau.edu.cn (Y.W.); 202430377@stu.sxau.edu.cn (J.L.); wangjing315@sxau.edu.cn (J.W.); zangql@sxau.edu.cn (Q.Z.); liangyanping@sxau.edu.cn (Y.L.); tianruxia@sxau.edu.cn (R.T.); 2 National Key Laboratory of Crop Genetics & Germplasm Enhancement and Utilization, Nanjing Agricultural University, Nanjing 210095, China; 2021204020@stu.njau.edu.cn (E.L.); changweizh@njau.edu.cn (C.Z.); jfl@njau.edu.cn (F.J.)

**Keywords:** *Capsicum annuum*, genome-wide association analysis, trait, SNP50K

## Abstract

As one of the most important vegetables globally, peppers have garnered significant attention from breeders due to their diverse agronomic traits, including plant type, leaf shape, and maturity. Understanding the genetic mechanisms underlying these traits is crucial for systematic advancements in sweet pepper breeding. In this study, leveraging the PepperSNP50K liquid breeding chip, we conducted a comprehensive analysis of horticultural traits and genetic diversity using sweet pepper germplasm samples. Initially, the sweet pepper populations were analyzed using SNP-based liquid chip technology. Subsequently, phenotypic surveys were performed on 217 sweet pepper samples, and the collected phenotypic data were integrated with SNP markers to conduct a genome-wide association study (GWAS) of key agronomic traits. Among the 25 horticultural traits evaluated, 11 exhibited significant associations with 54 SNP polymerization regions and 193 candidate genes. These findings provide a robust foundation for the utilization of sweet pepper germplasm resources and the development of new, improved varieties. Furthermore, in this study, we identified Caz06g05770 (Lycopene beta-cyclase) as a candidate gene responsible for the color of mature ripe fruits. This research not only enhances our understanding of the genetic basis of sweet pepper traits but also offers a practical roadmap for advancing breeding programs and boosting agricultural productivity. By bridging the gap between genetic research and practical breeding applications, this study paves the way for the development of high-yield, high-quality sweet pepper varieties tailored to meet the growing demands of global agriculture.

## 1. Introduction

In recent years, advancements in genetic research and biotechnological innovations have established genome-based breeding as an efficient and practical approach to rapidly address the need for diverse crop traits [1]. Genomic breeding strategies integrate principles from genetics, functional genomics, and phenotypic data, employing high-throughput DNA polymorphism detection to identify target genes and explore possible non-target genes associated with specific traits [2,3,4]. This approach offers an in-depth understanding of genomes, providing scientific evidence that informs agricultural targets and significantly enhancing the efficiency and accuracy of breeding selection [5]. At the core of such breeding lies genomic selection (GS), which relies on whole-genome marker-assisted selection. Current high-throughput genotyping technologies employed in genomic breeding include DNA sequencing and DNA microarrays [6,7]. With continuous technological advancements, next-generation sequencing (NGS) has gained widespread recognition as a genotyping method, owing to its high throughput, flexibility, and efficiency [8,9,10]. However, despite its advantages, NGS presents several limitations. Particularly in low-coverage sequencing, base data on single-nucleotide polymorphisms (SNPs) can be difficult to compare directly across different samples. Additionally, NGS data often require specialized bioinformatics expertise for analysis and processing, and the application of bioinformatics tools and resources in agriculture remains in its developmental stages [11]. These challenges significantly limit the scalability of NGS for large-scale crop breeding, particularly in the context of molecular breeding.

In comparison, DNA array technology (commonly referred to as DNA chips) offers a more cost-effective solution for genotyping. These arrays consist of a collection of microscopic DNA probes immobilized on a solid surface, enabling the simultaneous detection of thousands of genetic markers or the analysis of multiple genomic regions [12]. The most widely utilized SNP chip platforms are Illumina Infinium and Affymetrix Axiom. While these share similarities in terms of array formats, sizes, and applications, they exhibit notable differences in probe lengths, hybridization strategies, and SNP detection methods [13,14]. Compared to NGS, fixed SNP-based chips enable high-throughput genotyping more cost-effectively. Additionally, these chips can be customized for specific breeding targets, focusing on high-value functional alleles, thereby further optimizing breeding efficiency. Although SNP densities in arrays are typically lower than those detected through NGS, they are optimized to better suit specific breeding applications [9,15]. SNP-based breeding chips have been successfully applied in several major crops. For example, in rice, various chips have been widely employed for diverse purposes such as germplasm resource screening, varietal purity identification, and genetic background analysis, including Illumina Infinium Rice6K [16], Cornell_6K_Array_Infinium_Rice [17], Affymetrix Gene Chip Rice 44K [18], and Illumina Infinium RiceSNP50 [19]. In maize, the Illumina Infinium MaizeSNP50 chip was utilized for genetic analysis of two recombinant inbred line populations, resulting in the construction of high-density genetic linkage maps containing 20,913 and 14,524 markers, respectively [20]. Similarly, in soybean domestication studies, the Affymetrix Axiom NJAU 355KSoySNP chip was employed to analyze 105 wild and 262 cultivated accessions, verifying the domestication origin of soybean in central and northern China [10]. SNP chips have also been extensively applied in wheat breeding. For instance, the 90K Infinium iSelect SNP chip was used to scan four wheat populations, generating high-density genetic linkage maps with 29,692 SNP markers mapped to chromosomes, covering all 21 chromosomes of hexaploid wheat [21]. In more recent studies, the 660K wheat SNP chip demonstrated superior performance and was proven to outperform six previously available arrays, providing more comprehensive genome coverage [22]. In conclusion, SNP-based genotyping technologies, particularly DNA microarray platforms, have laid a strong foundation for analyzing genetic diversity, identifying key genomic loci, and facilitating molecular breeding in a wide range of crops. These tools have revolutionized traditional breeding methods, enabling breeders to accelerate the development of improved varieties with higher yield, superior quality, and enhanced adaptability.

Pepper (*Capsicum* spp.) belongs to the Solanaceae family and is an annual or perennial plant and a globally important vegetable and spice. Its fruits are rich in vitamin C and are also used as raw materials for extracting capsaicin and capsanthin, which have significant economic value [2,23,24,25,26]. Given the agricultural industry’s demand for sweet pepper varieties and breeding objectives, it is necessary to conduct genetic research on horticultural traits such as sweet pepper yield and quality, whose enhancement is influenced by characteristics including fruit length and width, flower number per axil, single fruit weight, the fruit shape index, vitamin C, and capsaicin content [2,27,28]. GWAS can identify QTLs and predict candidate genes for these and additional key agronomic traits in pepper, such as resistance and mechanical harvestability. The recently reported PepperSNP50K established a system conducive to the rapid breeding of sweet peppers via developing fertility restoration genes matching sterile lines and verifying their functions [23]. This method holds great promise for achieving the efficient molecular breeding of peppers.

In this study, we utilized sweet pepper germplasm materials and the PepperSNP50K chip to investigate genetic diversity and conduct a genome-wide association study (GWAS) of key horticultural traits. Phenotypic and genotypic data integration revealed 54 SNP loci significantly associated with 11 out of the 25 evaluated characteristics, including single fruit weight and fruit color, alongside 193 candidate genes. Caz06g05770 (Lycopene beta-cyclase) was further identified as a key gene regulating ripe fruit color. This research deepens the understanding of sweet pepper genetic traits and provides actionable molecular markers for breeding, bridging genetic advancements and practical applications and offering a roadmap for developing high-yield, high-quality varieties through molecular breeding. By leveraging these insights, breeders can accelerate the creation of improved sweet pepper cultivars to meet global agricultural demands, enhancing both productivity and resource utilization.

## 2. Results

### 2.1. Descriptive Statistical Analysis of Phenotypes of 217 Sweet Pepper Genotypes

**Figure 1:**
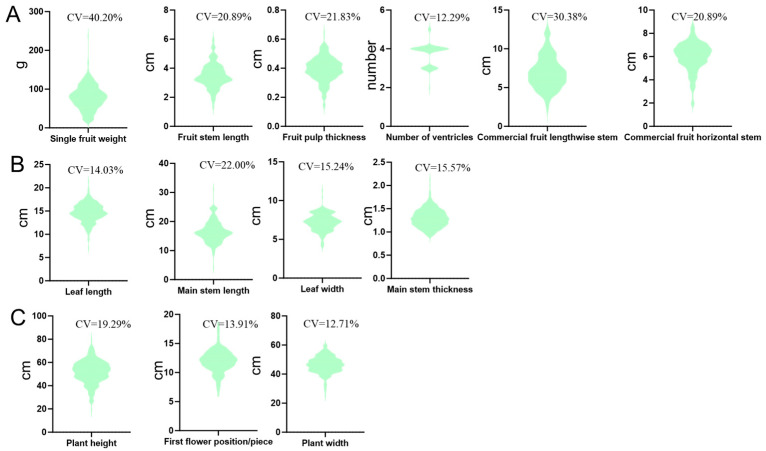
Descriptive statistical analysis of phenotypes in 217 sweet pepper genotypes. (**A**) Phenotypic distribution of fruit-related traits. (**B**) Phenotypic variation in leaf- and stem-related traits. (**C**) Phenotypic analysis of plant type-related traits. CV: coefficient of variation.

In 2022, a comprehensive survey was conducted to evaluate a total of 25 horticultural traits in 217 sweet pepper germplasm samples (Supplementary Tables S1, S2 and S5). These included key characteristics such as plant height, leaf shape, and anther color, which are essential for understanding the phenotypic diversity and agronomic potential of sweet pepper accessions. Among these traits, 13 quantitative traits were analyzed in detail and are presented in Figure 1.

These 13 characteristics were categorized into three major groups based on their relevance to different plant structures: fruit-related, plant type-related, and leaf- and stem-related traits. Fruit-related traits included metrics such as fruit length, width, and weight, which are directly associated with yield and marketability. Plant type-related traits, such as plant height and branching habits, reflected overall growth patterns and the adaptability of accessions to various cultivation environments. Finally, leaf- and stem-related traits included leaf length and width, which impact photosynthetic efficiency and plant health. These detailed phenotypic data provided a comprehensive resource for characterizing the genetic diversity of sweet pepper and served as a critical foundation for the subsequent GWAS (genome-wide association study) to explore the genetic basis of these important horticultural attributes.

**Figure 2:**
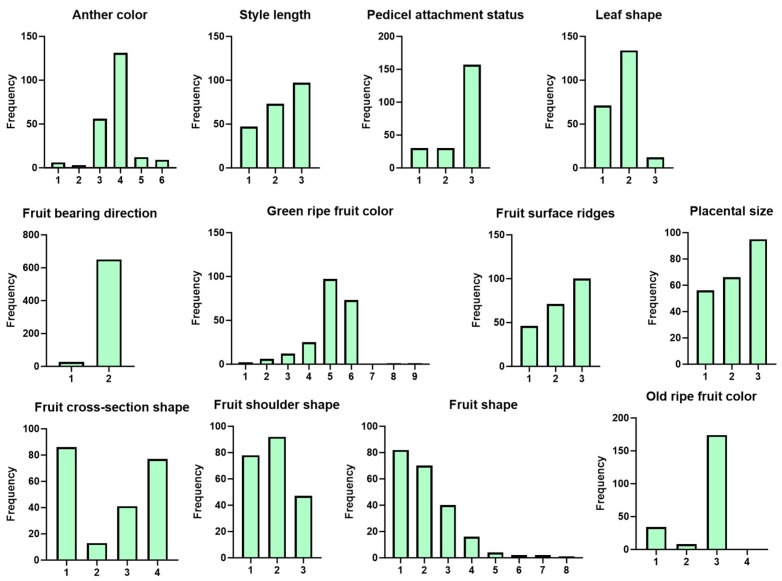
Descriptive statistical analysis of qualitative traits of 217 sweet pepper genotypes.

In addition to these quantitative traits, qualitative aspects were also investigated across the 217 sweet pepper accessions, such as anther color, fruit color, and fruit shape. These were carefully recorded and analyzed in order to understand their distribution and variation within the population. Their frequency distributions among the germplasm samples are illustrated in Figure 2 providing an overview of their occurrence and variability. For example, fruit color and shape, which are critical for consumer preference and market value, showed notable diversity across the population, reflecting the wide adaptation and genetic differentiation within these sweet pepper accessions.

This comprehensive phenotypic survey not only highlights the extensive morphological and horticultural diversity present in the 217 sweet pepper germplasm samples but also provides a valuable dataset for further genetic and breeding studies. The combination of quantitative and qualitative analyses forms a solid basis for identifying trait-associated genetic markers through GWAS. These efforts will ultimately contribute to the genetic improvement of sweet pepper by facilitating marker-assisted breeding and the development of improved varieties tailored to specific horticultural and market demands.

### 2.2. Population Analysis of 217 Sweet Pepper Germplasm Samples

**Figure 3:**
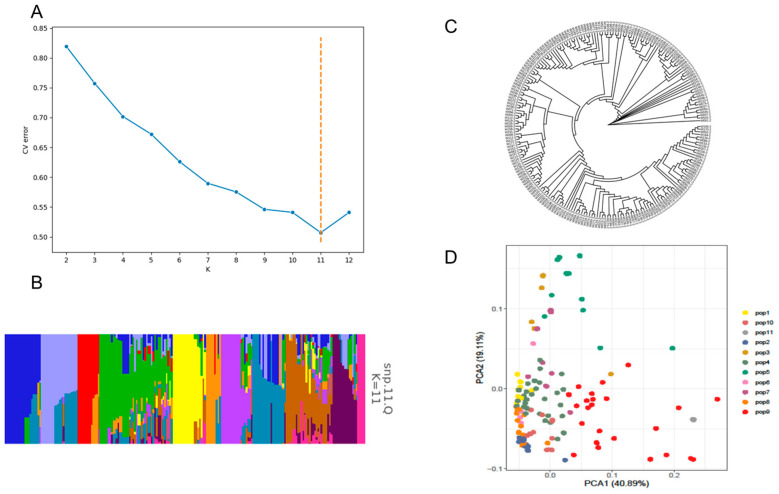
Population analysis of 217 sweet pepper germplasm samples. (**A**) ADMIXTURE was used for population structure assessment. (**B**) Population structure of sweet pepper accessions. (**C**) Phylogenetic tree of sweet pepper accessions based on SNP markers using NJ clustering method. (**D**) Principal component analysis of sweet pepper germplasm species.

To gain a deeper understanding of the genetic population structure of the 217 sweet pepper genotypes, we employed ADMIXTURE software to structurally analyze and subdivide them using SNP data obtained using the PepperSNP50K liquid-phase breeding chip. By analyzing the genetic composition of the samples, we aimed to clarify the levels of stratification and subgrouping present in this diverse resource pool. The results of the ADMIXTURE analysis revealed that when K = 11, the cross-validation (CV) error value reached its minimum, indicating optimal population stratification and providing strong evidence of a well-defined hierarchical structure (Figure 3A). This optimal K value suggests that the genetic diversity of the sweet pepper samples can be best described by dividing the population into 11 distinct subgroups (Figure 3B), each of which represents unique genetic clusters within the germplasm, reflecting the diverse evolutionary paths and breeding histories of these resources.

To further validate this population structure and provide supporting insights into the genetic relationships among the 217 genotypes, we constructed a neighbor-joining (NJ) evolutionary tree and utilized LD (linkage disequilibrium) decay (Supplementary Figure S1) and principal component analysis (Figure 3C,D). The resulting phylogenetic assessment confirmed the stratification observed in the ADMIXTURE evaluation, as the evolutionary tree also clearly divided the accessions into 11 discrete subgroups. The congruence between the ADMIXTURE results and the NJ tree underscores the robustness and reliability of these findings, highlighting the distinct genetic differentiation and evolutionary relationships among the sweet pepper genotypes.

The subdivision of this population into 11 genetic clusters offers valuable insights into the organization of sweet pepper diversity and serves as a foundation for further targeted analyses. Understanding the structure of the population has important implications for sweet pepper breeding strategies, as it allows for more efficient germplasm utilization, facilitates the identification of genetic resources carrying desirable traits, and provides a basis for exploring the evolutionary dynamics underlying sweet pepper domestication and genetic improvement.

### 2.3. Whole-Genome Association Analysis of Sweet Pepper Traits

In this study, we performed a comprehensive association analysis by linking the survey results of 25 horticultural traits from 217 sweet pepper accessions with SNP data obtained through the PepperSNP50K liquid-phase breeding chip. Among the 25 characteristics evaluated, 11 were found to have significant associations with 54 genomic loci, while no significant associations were observed for the remaining 15 traits (Table 1 and Supplementary Table S3). This suggests that some traits may be controlled by complex genetic architectures or environmental interactions that were not fully captured in this study.

The 11 traits that exhibited significant associations included a variety of horticulturally important aspects, such as anther color, the longitudinal and transverse diameters of commercial fruits, fruit shape, fruit grooves and clefts, immature (green) color, ripe fruit color, the number of locules, leaf length, attachment status, and single fruit weight (Supplementary Figures S2–S12). Collectively, these traits were associated with a total of 45 significant SNP loci, underscoring their genetic basis and their potential in future marker-assisted breeding programs. The identification of these loci provides important insights into the genetic control mechanisms governing such traits and serves as a valuable resource for advancing molecular breeding in sweet peppers.

**Figure 4:**
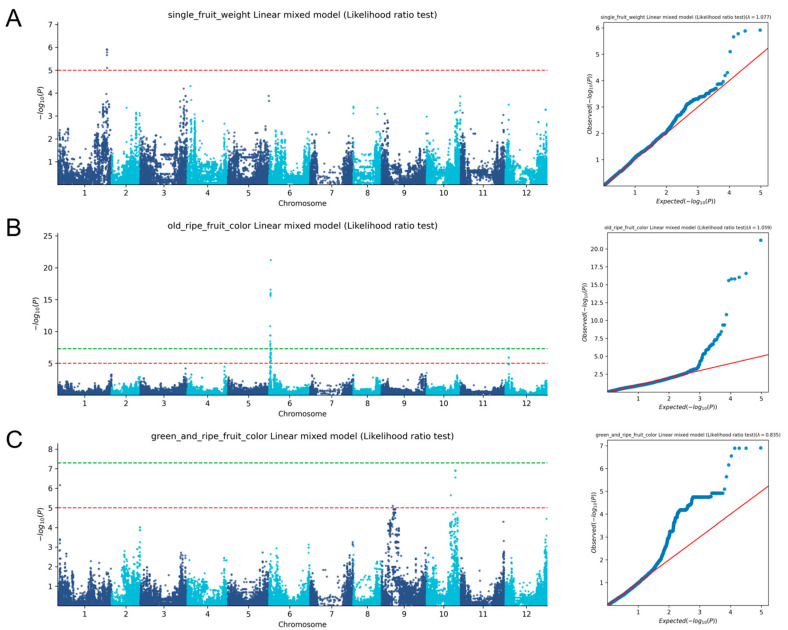
Single fruit weight- and fruit color-related loci associated with GWAS based on Pepper50K. (**A**) Significant loci associated with single fruit weight. (**B**) Significant loci associated with mature ripe fruit color. (**C**) Significant loci associated with green and ripe fruit color. The red arrow represents a more relaxed threshold of *p* < 1 × 10^−5^ in order to further investigate potential weak associations, and the green arrow represents a stringent threshold of *p* < 5 × 10^−8^ for identifying strongly associated SNPs.

Given the economic and agricultural significance of sweet pepper fruits, we particularly focused on the GWAS results for traits related to fruits. As shown in Figure 4, the analysis revealed strong genetic signals for single fruit weight, immature (green) fruit color, and ripe (mature) fruit color, indicating the presence of significant genomic regions contributing to the variability in these traits. For single fruit weight, a key determinant of yield and market value, a total of four significant SNP loci were identified. Notably, all four were clustered on chromosome Chr01, suggesting a major genetic region responsible for controlling fruit weight.

Meanwhile, a total of four SNP loci were found to be significantly associated with immature fruit color, another trait important for early harvest and market preferences. These were distributed across chromosomes Chr01 and Chr10, reflecting the involvement of multiple genomic regions in determining immature fruit color. Furthermore, the genetic analysis of ripe fruit color, which greatly influences consumer acceptance and quality, uncovered that 12 SNP loci were significantly associated with this trait. Interestingly, all of these loci were located on chromosome Chr06, suggesting that it contains candidate genes that play a pivotal role in defining ripe fruit color in sweet peppers.

The identification of these significant SNP loci provides new opportunities for understanding the genetic determinants underlying horticultural traits in sweet peppers and lays the groundwork for further research on the functional genes responsible for these traits. Additionally, these findings have practical implications for sweet pepper breeding, allowing for the more precise manipulation of key characteristics using marker-assisted selection. By incorporating these SNP markers into breeding programs, it may be possible to accelerate the development of new sweet pepper varieties with improved yield, quality, and marketability while meeting the diverse needs of growers and consumers.

### 2.4. Genome-Wide Association Study (GWAS)-Identified Candidate Genes

Based on the results of the single-phenotype GWAS and the significantly associated loci identified in this study, we combined gene annotation data from the reference genome with existing research findings to select candidate genes associated with important agronomic traits in sweet pepper. The selected genes are listed by chromosome location, and detailed functional annotations are provided (Supplementary Table S4). These findings provide valuable new insights into the genetic and molecular mechanisms underlying the development of plant architecture and other key agronomic traits in sweet pepper.

Among the characteristics analyzed, single fruit weight is of particular interest, as it is one of the most critical factors influencing sweet pepper yield, a core breeding objective. Similarly, fruit color plays a pivotal role in consumer preferences and marketability, making it another important target for genetic improvement. Given the agronomic and economic importance of these traits, in this study, we focused on identifying candidate genes associated with them.

For single fruit weight, the GWAS revealed eight significant candidate genes, all localized on chromosome 1 (Chr01). This result aligns with previous studies that identified this locus as a major region controlling fruit weight in sweet pepper varieties [28]. The discovery of these candidate genes reinforces the importance of Chr01 in regulating fruit weight and provides new genetic targets for improving yield in sweet pepper breeding programs.

For mature fruit color, the GWAS identified 12 significant SNPs, all located on chromosome 6 (Chr06). Among these associated loci was the candidate gene Caz06g05770, which encodes Lycopene beta-cyclase (LCYB), a key enzyme in the carotenoid biosynthesis pathway that plays a crucial catalytic role in converting Lycopene into beta-carotene, an essential precursor of carotenoids that determine fruit coloration. This finding highlights Caz06g05770 as a key genetic locus involved in sweet pepper fruit pigmentation. Its functional role in the carotenoid synthesis pathway suggests that it may serve as an important target for engineering or selecting desired fruit color phenotypes in sweet pepper breeding.

## 3. Discussion

As a globally cultivated and important economic and edible crop, sweet pepper is favored by consumers worldwide for its unique flavor, rich nutritional value, and extensive applications in the food industry [29,30]. Based on the agricultural demand for sweet pepper varieties and breeding goals, it is highly essential to conduct genetic research on horticultural traits related to yield and quality [31,32]. Although previous studies have made significant progress in the mapping of such characteristics, the exploration of significant genes has been limited to individual samples with relatively low analytical efficiency. By employing the GWAS (genome-wide association study) approach, it is possible to utilize unrelated natural populations while analyzing multi-year and multi-location data for a variety of traits [2,10,27,28,33,34]. Additionally, the development of a large number of genetic markers significantly improves the accuracy of trait mapping, even allowing for the identification of single genes. This study provides a comprehensive analysis of genetic and phenotypic diversity in sweet pepper germplasm samples. By leveraging population structure analysis, phenotypic surveys, genome-wide linkage disequilibrium (LD) decay mapping, and GWAS, we identified significant loci and candidate genes associated with critical traits such as fruit weight and color. More importantly, it was demonstrated that SNP50K can provide practical value in breeding programs that improve yield, quality, and market adaptability.

Our analysis of the population structure of 217 sweet pepper germplasm accessions revealed 11 distinct genetic subgroups, confirmed by ADMIXTURE, phylogenetic tree clustering (NJ method), and principal component analysis (PCA). This well-defined stratification reflects the evolutionary trajectories and breeding histories of these accessions, as well as the organization of their genetic diversity, which has important implications for breeding strategies. Breeders can explore genetic clusters to identify highly differentiated resources, expand breeding pools, and rapidly introgress desirable traits into elite lines. By better understanding population stratification, germplasm resources can be more efficiently utilized, accelerating trait improvement for specific market and cultivation demands.

The phenotypic evaluation of the 217 sweet pepper genotypes demonstrated extensive variability in traits related to fruits, such as their weight, color, and shape, as well as plant type and leaf/stem characteristics. These results indicate high horticultural and ecological adaptability within the germplasm, reflecting its diverse origins. Variations in quantitative traits (e.g., fruit length and plant height) and qualitative traits (e.g., anther color and fruit shape) represent valuable sources of genetic diversity for breeding programs. The recorded phenotypic data act as a critical resource for correlating traits with genetic loci through GWAS. Using GWAS, we identified 54 significant loci associated with 11 agronomically and economically important horticultural traits, highlighting opportunities for sweet pepper genetic improvement and providing essential references for dissecting complex genetic architectures controlling various characteristics.

Single fruit weight is a key determinant of yield and an important trait in breeding programs. Our GWAS results identified eight significant SNPs associated with this trait, all localized on chromosome 1 (Chr01). In another report on the GWAS of sweet pepper characteristics, single fruit weight also showed a clear signal on Chr01 [28]. The consistency between our results and earlier studies highlights the robustness of GWAS for identifying yield-associated loci. These genes serve as targets for developing high-yield sweet pepper varieties, demonstrating the potential of molecular breeding to address production demands.

The mature fruit color of sweet pepper is an important indicator of its commercial quality. Early genetic studies on sweet pepper fruit suggested that mature fruit color is controlled by a single gene, denoted as y, where y⁺ represents red (dominant) and y represents yellow [35,36,37]. Although the theory that the mature fruit color of sweet peppers is controlled by three independent gene pairs has been widely recognized, its genotypes and phenotypes do not fully conform to the genetic model proposed [38,39]. The significant differences in the genetic research on the mature fruit color of sweet peppers among domestic and international scholars may be attributed to the developmental stages of the fruit, the complexity of the fruit’s color composition, and subjective human determination of color differences. Further, they may be due to the diversity of sweet pepper germplasm resources, which determines the variability in and complexity of the genetic basis of fruit color and can result in different genetic backgrounds in hybrid populations and ultimately affect the segregation of mature fruit colors. Even sweet peppers with seemingly identical fruit colors when judged visually may contain different types and amounts of carotenoids, and their genotypes may also differ. Our analysis revealed 12 significant loci associated with mature fruit color, all located on chromosome 6 (Chr06). Notably, one of the key candidate genes identified was Caz06g05770, which encodes Lycopene beta-cyclase (LCYB). LCYB is a pivotal enzyme in the carotenoid biosynthesis pathway and catalyzes the conversion of Lycopene to beta-carotene, a precursor of carotenoids that affect coloration. Silencing the LCYC gene in peppers led to a noticeable change in fruit coloration [40]. Similarly, a 2 bp insertion in the LCYC gene of papaya was associated with distinct variations in fruit pigmentation [41]. Our finding aligns with the known biochemical pathways of pigment production and highlights Caz06g05770 as a critical target for regulating fruit color in sweet peppers.

## 4. Materials and Methods

### 4.1. Plant Materials and DNA Extract

To validate the performance of the pepper GBTS gene chip, we selected 217 sweet pepper germplasm samples, whose origins are documented in Supplementary Table S1. All sweet pepper plants were cultivated in the Base of the Horticulture College, Shanxi Agricultural University. Leaves were collected 60 days after sowing for sampling, and DNA was extracted from the plants using the CTAB method [42]. The quality of the DNA samples was subsequently assessed using a Nano Drop3000 and agarose gel electrophoresis.

### 4.2. Agronomic Trait Survey

The phenotypic investigation of the sown materials was conducted three times. The seedlings were first raised in a greenhouse in Jinzhong, Shanxi, and then transplanted to an artificial intelligence-controlled greenhouse for final planting. We investigated 25 agronomic traits in sweet pepper, including anther color, pedicle growth status, leaf shape, green and ripe fruit color, fruit surface ridges, fruit shape, mature ripe fruit color, leaf length, commercial fruit lengthwise stem, commercial fruit horizontal stem, the number of ventricles, and single fruit weight, each of which was categorized as either qualitative or quantitative and surveyed at least three times, with 5–8 replicated plant lines per observation. Descriptive statistics (e.g., mode, mean, and max.) were calculated and graphs were drawn in Microsoft Excel 2007. The results were presented as the mean of nine replications.

### 4.3. Genotyping Using PepperSNP50K Liquid-Phase Breeding Chip

In this study, we utilized liquid-phase breeding microarray data on peppers, sourced from Hunan Agricultural University [23]. DNA was isolated from mixed pools of fertile and infertile pepper plants, each containing over 30 individual specimens. A modified protocol compatible with MGI’s genomic sequencing platform was employed to prepare whole-genome fragment libraries. Briefly, 500 ng of pepper genomic DNA was fragmented into 200–300 bp segments. Adapters suitable for the DNBSEQ-T7 platform were ligated following end repair and 3′-A tailing. The resulting libraries were hybridized with probes designed for the Pepper50K liquid-phase breeding microarray. Biotinylated target-specific fragments were selectively bound to their complementary sequences and captured using streptavidin-coated magnetic beads. The enriched DNA fragments underwent PCR amplification and circularization for sequencing on the DNBSEQ-T7 platform (MGI, Shenzhen, China) with a PE150 module, with a sequencing depth of ~100× per target region achieved for accuracy. The raw reads were filtered using established criteria, and VCF tools were applied to refine SNP loci on chromosomes without deletions or dimorphism for BSA analysis (missing rate <= 0.1; heterozygosity rate <= 0.1; maf >= 0.05).Allele segregation was evaluated using the Euclidean distance formula, chosen for its independence from parental strain data and robustness against noise, based on SNP genotype and sequencing depth variations across the pools [43].

### 4.4. Phylogenetic and Population Analyses

The high-quality single-nucleotide polymorphisms (SNPs) and small insertions–deletions (indels) we obtained were used for phylogenetic and population analysis. The ML phylogenetic tree was generated using SNPs with IQ-TREE (-m GTR+F+R6 -fast), employing the best model determined by the Bayesian information criterion. IQ-TREE bootstrap support values were calculated using the ultrafast bootstrap approach with 1000 replicates [44], and the phylogenetic tree was visualized using the online tool EvolView [45]. Principal component analysis was performed using Plink (v1.9) with the entire set of SNPs, and population structure analysis was conducted using STRUCTURE v2.3.4 [46]. The optimal K was determined by analyzing K values from 1 to 12 and selecting the one with the lowest cross-validation error (CV error). LD decay was calculated for all pairs of SNPs within 300 kb using PopLDdecay (v3.27) with the parameters ‘-MaxDist 300 -Het 0.1 -Miss 0.1′ [47].

### 4.5. Genome-Wide Association Study

The GWAS was performed using GEMMA software to identify significant SNP–trait associations [48]. High-quality SNPs were filtered with a minor allele frequency (MAF) > 0.01 and a call rate > 90%. A kinship matrix was calculated to account for relatedness, and a mixed linear model (MLM) was applied with this matrix as a random effect to control for population structure. Association tests were conducted for each SNP, and significance was assessed using the likelihood ratio test (LRT) and the Wald test, comparing the null model (no SNP effect) with the alternative model (SNP effect present). We calculated *p*-values for two significance thresholds in our GWAS and considered SNPs with values under these significant: a stringent threshold of *p* < 5 × 10^−8^ for identifying strongly associated SNPs and a more relaxed threshold of *p* < 1 × 10^−5^ to explore potential weak associations for further investigation. To correct for multiple testing, a false discovery rate (FDR) adjustment was applied. Significant SNPs were visualized using Manhattan and quantile–quantile (QQ) plots [49]. The former displayed the association of each SNP with phenotypes across the genome, highlighting regions with significant *p*-values, while the latter were used to assess the distribution of observed *p*-values against the expected uniform distribution, helping to identify potential deviations from the null hypothesis and evaluate the presence of false positives using R software (version 3.5.0).

### 4.6. Gene Annotation

The coding sequence (CDS) and genome annotation files of Zhangshugang were downloaded [33], and subsequently processed and formatted using TBtools (Ver.2.227) to ensure compatibility for downstream gene annotation analysis [50]. Significant SNPs identified in the GWAS were annotated using ANNOVAR (Ver.2020Jun08) [51], which provided functional annotations via mapping each SNP to genes, regulatory regions, and known databases, allowing for the interpretation of their potential biological roles and associations with phenotypes.

## 5. Conclusions

In this study, we analyzed genetic diversity and population structure based on the SNP50K chip combined with the phenotypical traits of 217 sweet peppers. A genome-wide association study (GWAS) evaluated 25 agronomic traits, in which it was found that 11 were significantly associated with 54 SNP aggregation regions and 193 candidate genes. These results lay the foundation for the utilization of sweet pepper germplasm resources and the development of new varieties.

## Figures and Tables

**Table 1:** Genome-wide significant associations with agronomic traits.

Trait	Number of Significantly Associated SNPs	Associated Chromosomes
Anther color	1	2
Commercial fruit horizontal	6	2, 3, 9, 11
Commercial fruit lengthwise	1	4
Fruit shape	1	1
Fruit surface ridges	2	3, 5
Green and ripe fruit color gene	4	1, 10
Leaf length	1	9
Number of ventricles	1	6
Old ripe fruit color gene	12	6
Pedicel attachment status	12	1, 3, 6, 8, 12
Single fruit weight	4	1

## Data Availability

The original contributions presented in this study are included in the article/Supplementary material. Further inquiries can be directed to the corresponding author.

## Supplementary Materials

The following supporting information can be downloaded at https://www.mdpi.com/article/10.3390/plants14101506/s1. Table S1: Sweet pepper germplasm information. Table S2: Phenotypic data for 217 sweet peppers. Table S3: SNP loci and genes associated with traits. Table S4: Annotation of candidate genes. Table S5: Qualitative trait standards. Figure S1: Linkage disequilibrium decay determined according to squared correlations of allele frequencies (R2). Figure S2: Pedicel attachment status related loci associated with GWAS based on peper50K and its phenotypic types. Figure S3: Fruit surface ridges related loci associated with GWAS based on peper50K and its phenotypic types. Figure S4: Green and ripe fruit color related loci associated with GWAS based on peper50K and its phenotypic types. Figure S5: Commercial fruit horizontal stem related loci associated with GWAS based on peper50K and its phenotypic types. Figure S6: Commercial fruit lengthwise stem related loci associated with GWAS based on peper50K and its phenotypic types. Figure S7: Fruit shape related loci associated with GWAS based on peper50K and its phenotypic types. Figure S8: Anther color related loci associated with GWAS based on peper50K and its phenotypic types. Figure S9: Number of ventricles related loci associated with GWAS based on peper50K and its phenotypic types. Figure S10: Leaf length related loci associated with GWAS based on peper50K and its phenotypic types. Figure S11: Old ripe fruit color related loci associated with GWAS based on peper50K and its phenotypic types. Figure S12: Single fruit weight related loci associated with GWAS based on peper50K and its phenotypic types.
