# Supplementary material for: Genome-Wide Association Analysis of Sweet Pepper (Capsicum annuum) Based on Agronomic Traits Using PepperSNP50K
Source: Plants (Basel). 2025 May 17;14(10):1506. doi: 10.3390/plants14101506 (PMC12114862; doi:10.3390/plants14101506)
Supplement: Supplementary file 1 [file plants-14-01506-s001.zip › Supplementary Figures.pdf]

Supplementary Note, Figures, Gif, and Tables

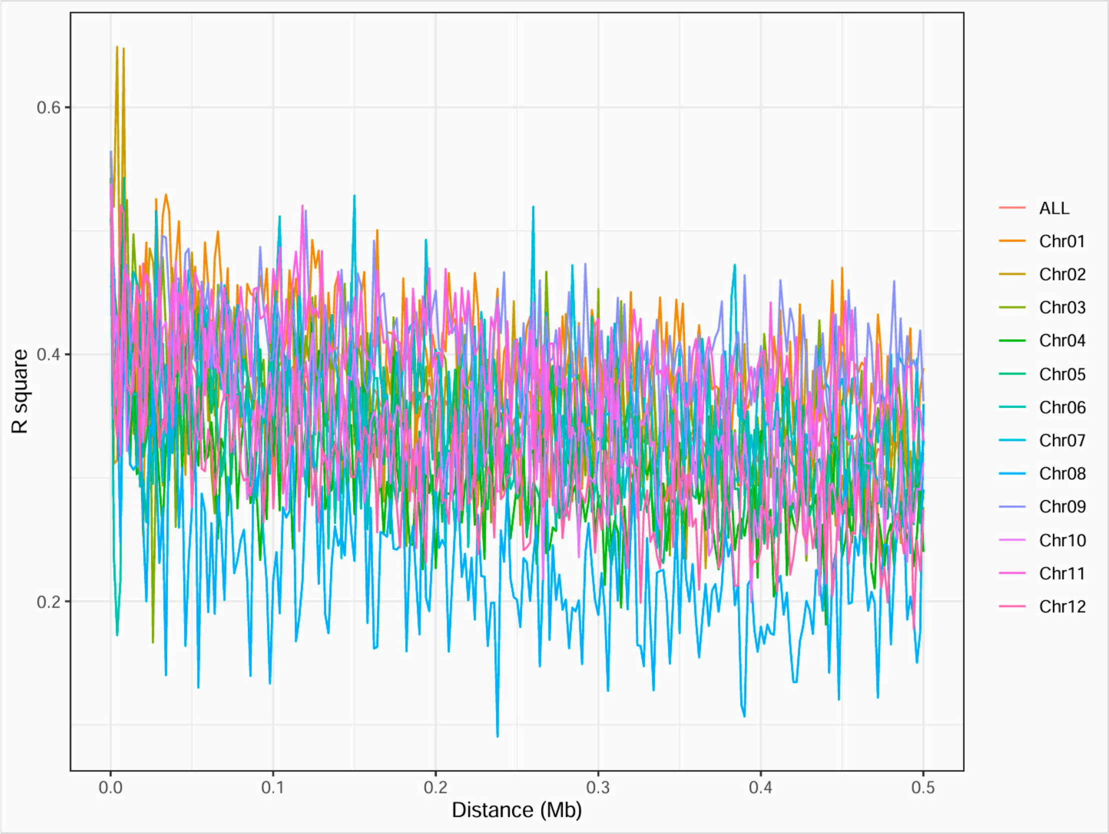

Supplementary Figure S1: Linkage disequilibrium decay determined according to squared correlations of allele frequencies (R^2).

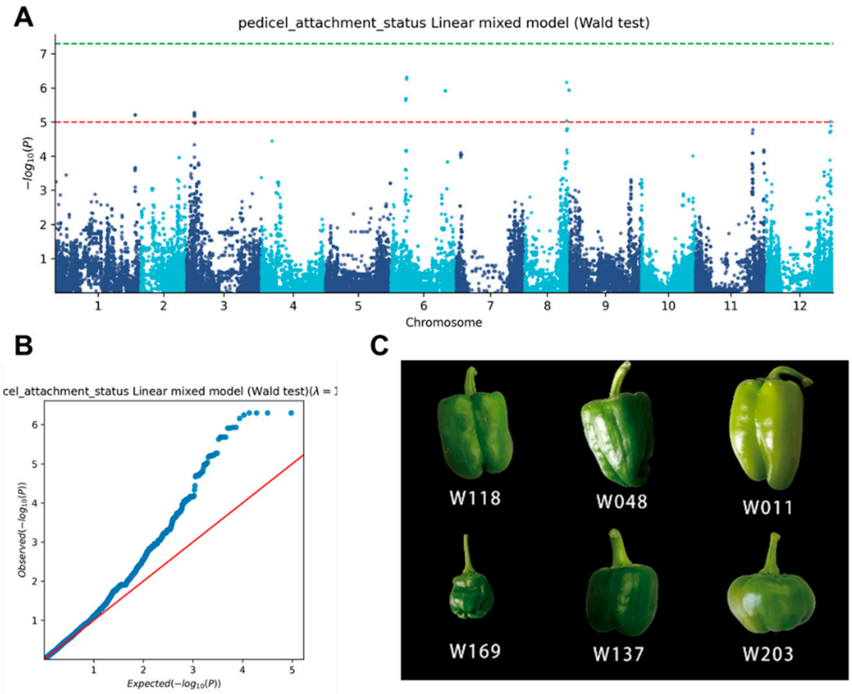

Supplementary Figure S2: Pedicel attachment status related loci associated with GWAS based on peper50K and its phenotypic types. (A) Significant loci associated with pedicel attachment status.

(B) Q-Q plots of pedicel attachment status. (C) Phenotypic types of pedicel attachment status.

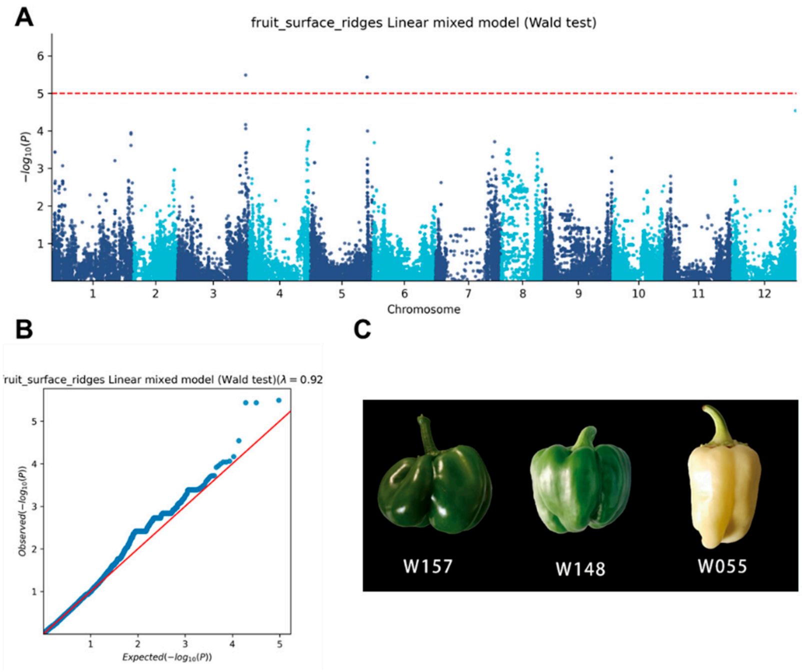

Supplementary Figure S3: Fruit surface ridges related loci associated with GWAS based on peper50K and its phenotypic types. (A) Significant loci associated with fruit surface ridges. (B) Q-Q plots of fruit surface ridges. (C) Phenotypic types of fruit surface ridges.

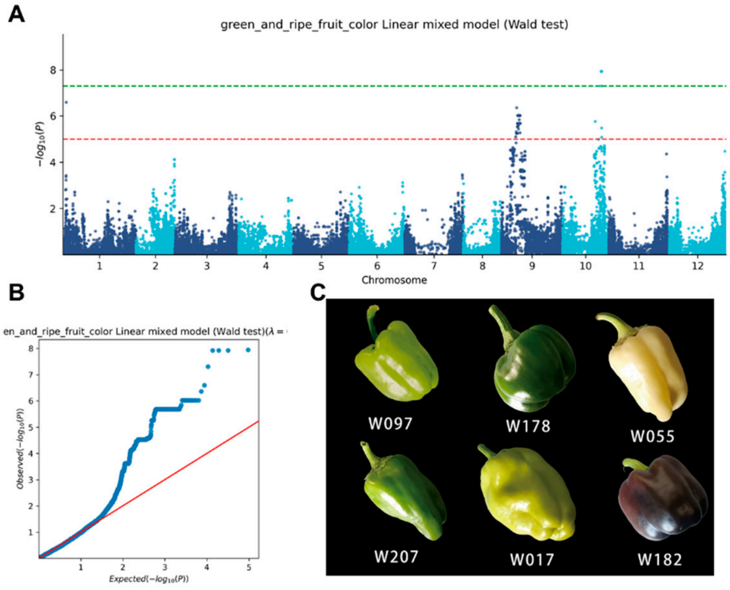

Supplementary Figure S4: Green and ripe fruit color related loci associated with GWAS based on peper50K and its phenotypic types. (A) Significant loci associated with green and ripe fruit color. (B) Q-Q plots of green and ripe fruit color. (C) Phenotypic types of green and ripe fruit color.

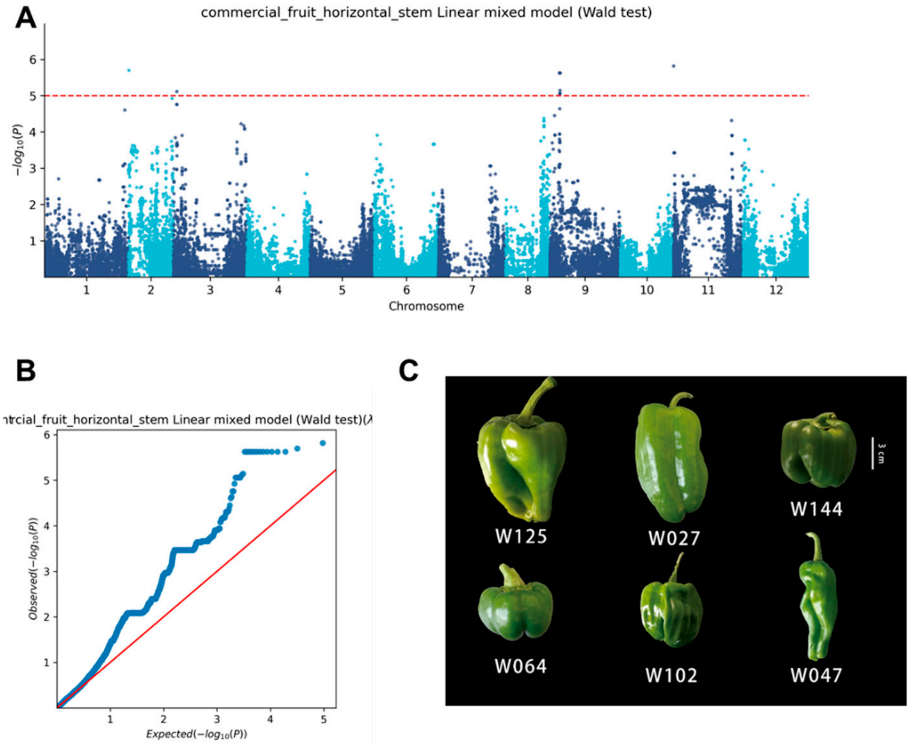

Supplementary Figure S5: Commercial fruit horizontal stem related loci associated with GWAS based on peper50K and its phenotypic types. (A) Significant loci associated with commercial fruit horizontal stem. (B) Q-Q plots of commercial fruit horizontal stem. (C) Phenotypic types of commercial fruit horizontal stem.

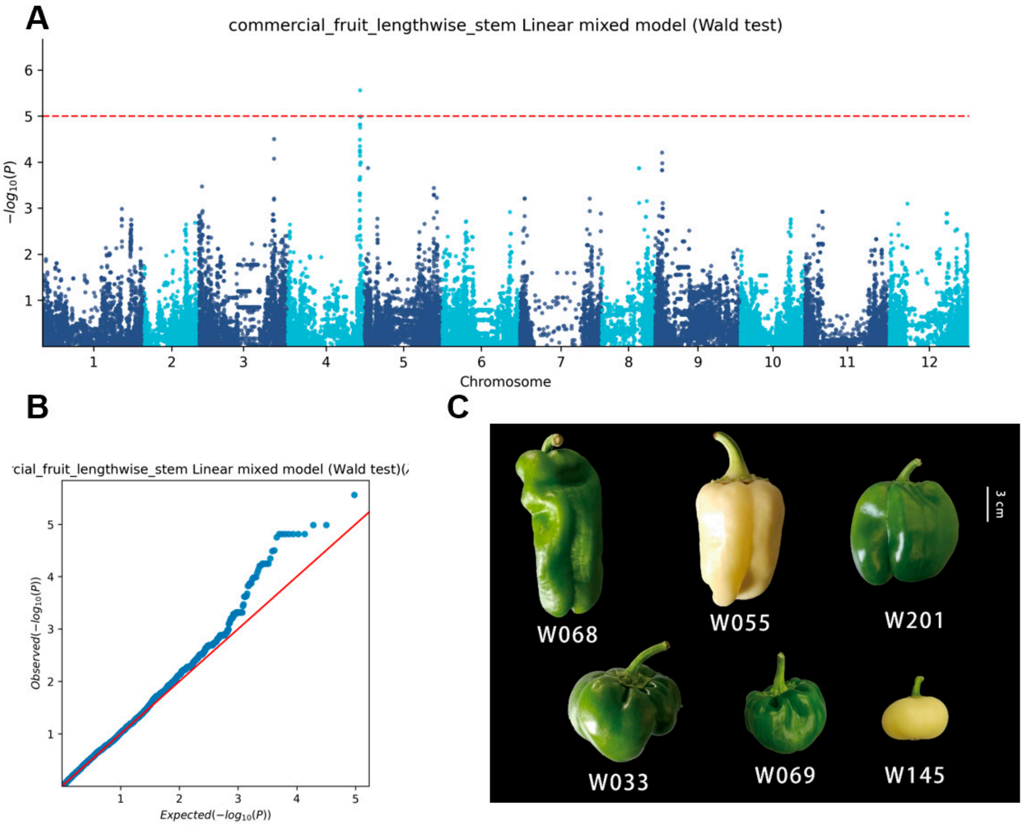

Supplementary Figure S6: Commercial fruit lengthwise stem related loci associated with GWAS based on peper50K and its phenotypic types. (A) Significant loci associated with commercial fruit lengthwise stem. (B) Q-Q plots of commercial fruit lengthwise stem. (C) Phenotypic types of commercial fruit lengthwise stem.

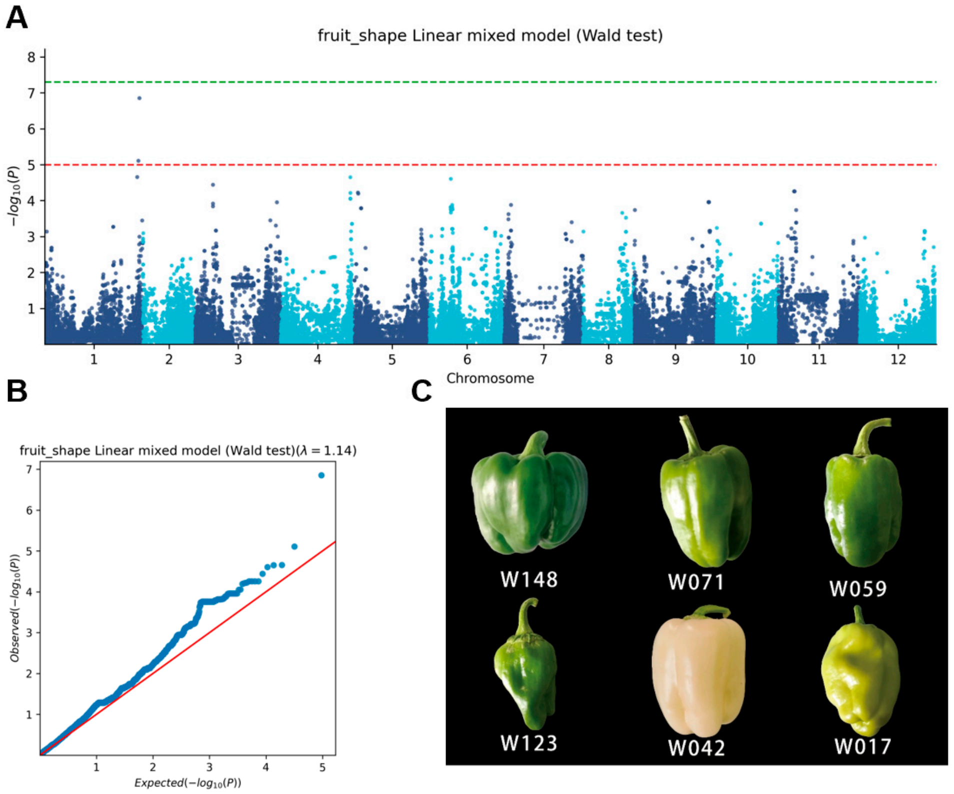

Supplementary Figure S7: Fruit shape related loci associated with GWAS based on peper50K and its phenotypic types. (A) Significant loci associated with fruit shape. (B) Q-Q plots of fruit shape. (C) Phenotypic types of fruit shape.

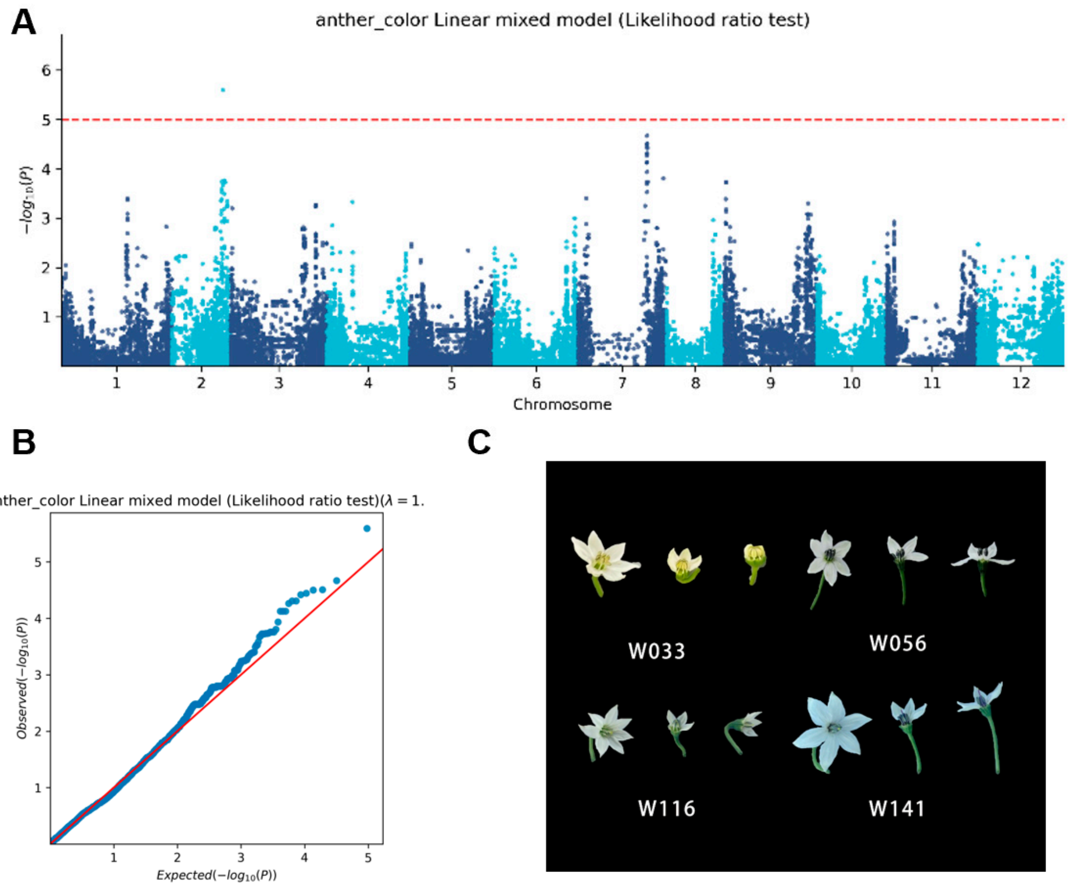

Supplementary Figure S8: Anther color related loci associated with GWAS based on peper50K and its phenotypic types. (A) Significant loci associated with anther color. (B) Q-Q plots of anther color. (C) Phenotypic types of anther color.

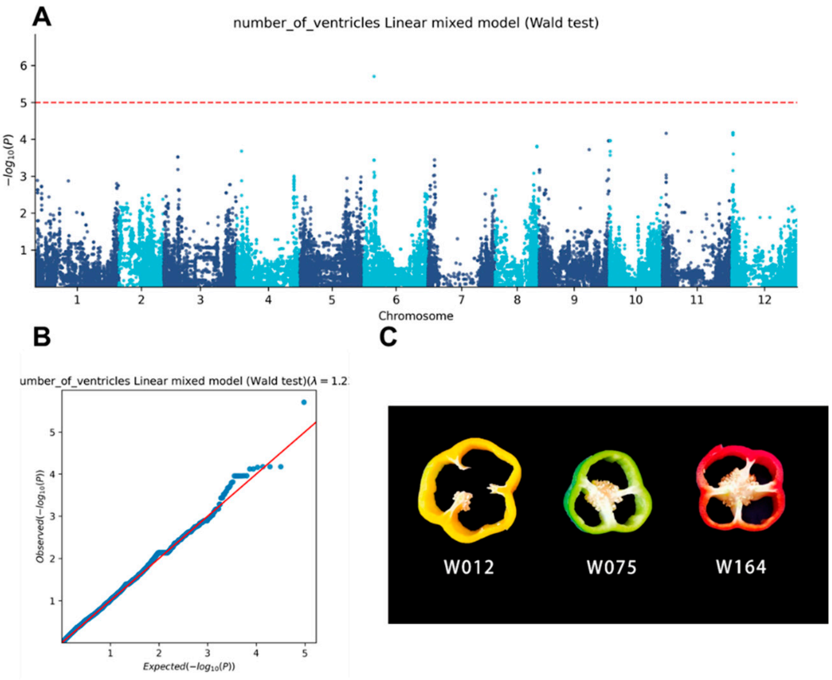

Supplementary Figure S9: Number of ventricles related loci associated with GWAS based on peper50K and its phenotypic types. (A) Significant loci associated with number of ventricles. (B) Q-Q plots of number of ventricles. (C) Phenotypic types of number of ventricles.

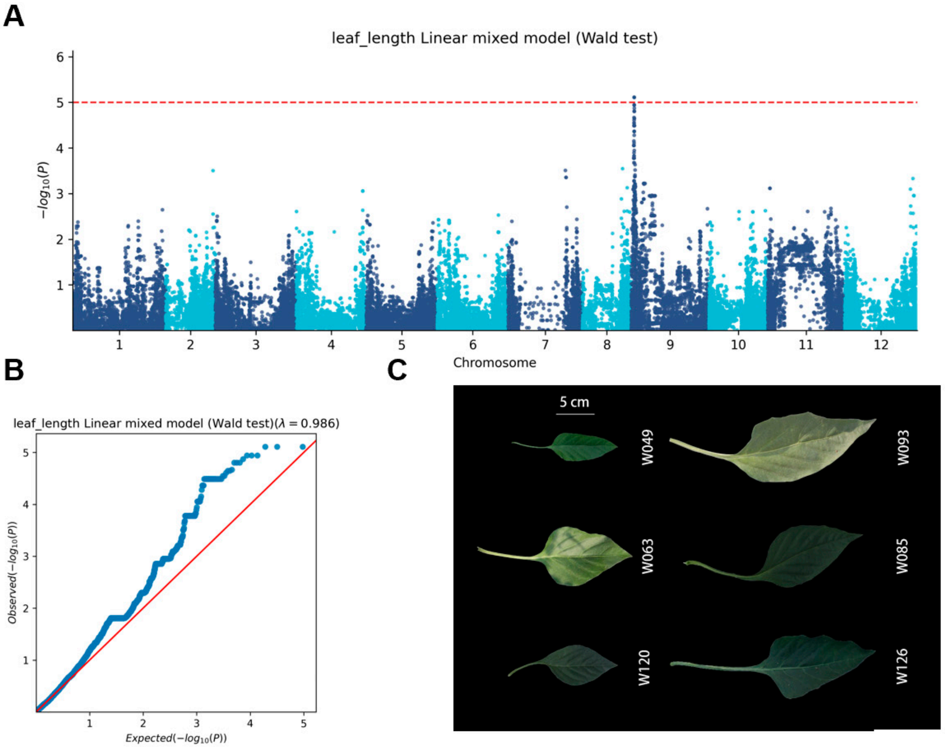

Supplementary Figure S10: Leaf length related loci associated with GWAS based on peper50K and its phenotypic types. (A) Significant loci associated with leaf length. (B) Q-Q plots of leaf length. (C) Phenotypic types of leaf length.

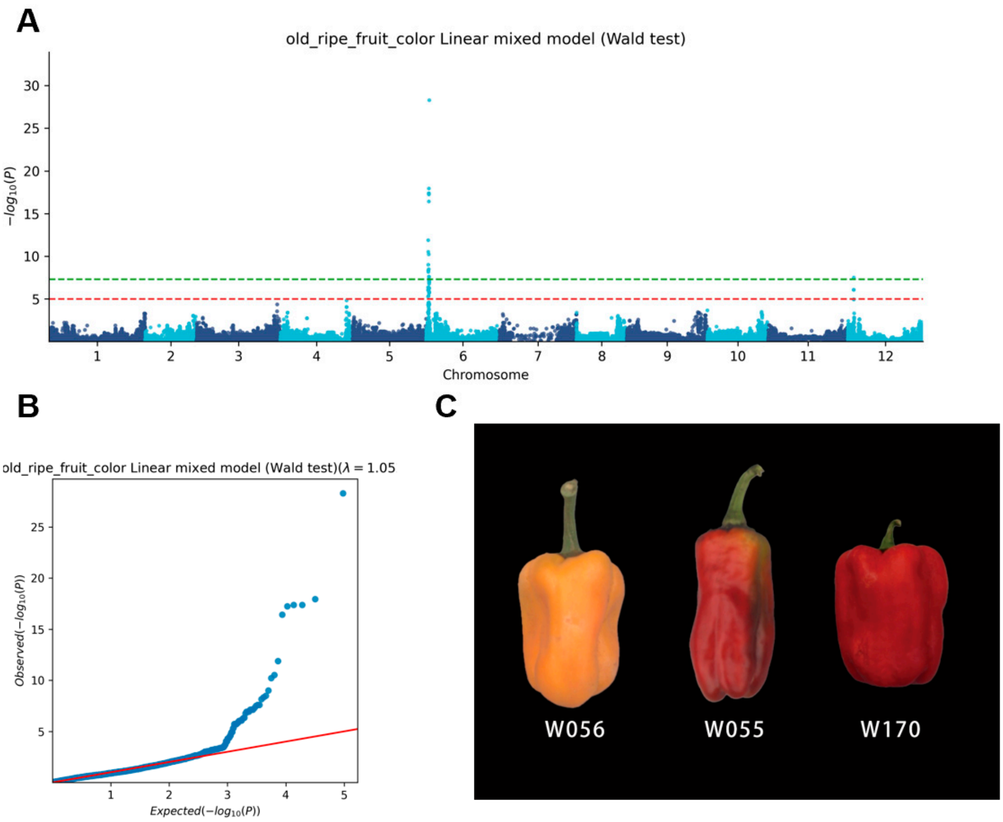

Supplementary Figure S11: Old ripe fruit color related loci associated with GWAS based on peper50K and its phenotypic types. (A) Significant loci associated with old ripe fruit color. (B) Q-Q plots of old ripe fruit color. (C) Phenotypic types of old ripe fruit color.

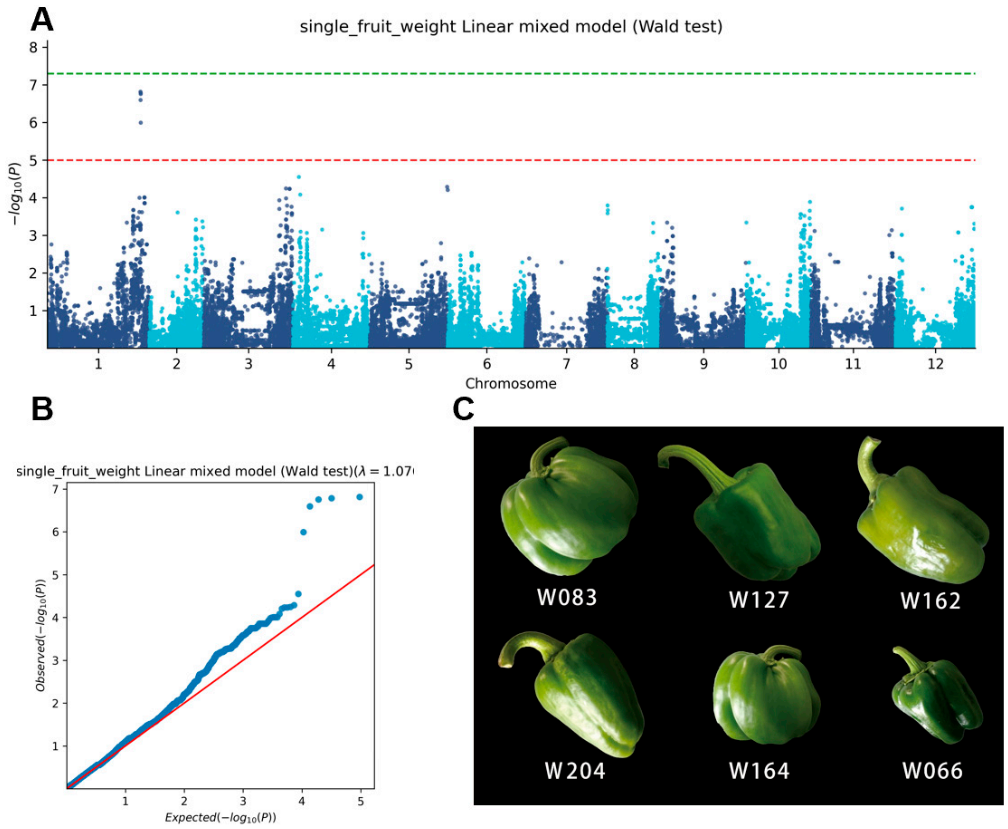

Supplementary Figure S12: Single fruit weight related loci associated with GWAS based on peper50K and its phenotypic types. (A) Significant loci associated with single fruit weight. (B) Q-Q plots of single fruit weight. (C) Phenotypic types of single fruit weight.
